# Phosphate Uptake from Phytate Due to Hyphae-Mediated Phytase Activity by Arbuscular Mycorrhizal Maize

**DOI:** 10.3389/fpls.2017.00684

**Published:** 2017-04-28

**Authors:** Xin-Xin Wang, Ellis Hoffland, Gu Feng, Thomas W. Kuyper

**Affiliations:** ^1^College of Resources and Environmental Sciences and Centre for Resources, Environment and Food Security, China Agricultural UniversityBeijing, China; ^2^Department of Soil Quality, Wageningen University and ResearchWageningen, Netherlands

**Keywords:** mycelium, phytase, acid phosphatase, maize cultivars, phytate

## Abstract

Phytate is the most abundant form of soil organic phosphorus (P). Increased P nutrition of arbuscular mycorrhizal plants derived from phytate has been repeatedly reported. Earlier studies assessed acid phosphatase rather than phytase as an indication of mycorrhizal fungi-mediated phytate use. We investigated the effect of mycorrhizal hyphae-mediated phytase activity on P uptake by maize. Two maize (*Zea mays* L.) cultivars, non-inoculated or inoculated with the arbuscular mycorrhizal fungi *Funneliformis mosseae* or *Claroideoglomus etunicatum*, were grown for 45 days in two-compartment rhizoboxes, containing a root compartment and a hyphal compartment. The soil in the hyphal compartment was supplemented with 20, 100, and 200 mg P kg^-1^ soil as calcium phytate. We measured activity of phytase and acid phosphatase in the hyphal compartment, hyphal length density, P uptake, and plant biomass. Our results showed: (1) phytate addition increased phytase and acid phosphatase activity, and resulted in larger P uptake and plant biomass; (2) increases in P uptake and biomass were correlated with phytase activity but not with acid phosphatase activity; (3) lower phytate addition rate increased, but higher addition rate decreased hyphal length density. We conclude that P from phytate can be taken up by arbuscular mycorrhizal plants and that phytase plays a more important role in mineralizing phytate than acid phosphatase.

## Introduction

Phosphorus (P) is an essential plant nutrient that limits agricultural production on many soils (Vance, 2001). Although the total amount of P in soils often exceeds plant demand, most P occurs in forms that are unavailable for plant uptake. Plants can take up only orthophosphate from the soil solution. Most of the P in soils, however, is not in the soil solution but is (strongly) adsorbed to mineral surfaces (metal oxides) and/or precipitated as poorly soluble phosphate salts. Also a large part of soil P (between 30 and 80%; Dalal, 1977) can be present as organic P. Organic P cannot be directly taken up by plants. Plants or soil microbes need to hydrolyze organic P, after which the orthophosphate can be taken up. However, the largest part of the organic P pool is also adsorbed or fixed and thereby inaccessible to these hydrolytic enzymes (Turner et al., 2002). Inositol phosphates constitute the largest component (over 60%; Dalal, 1977; Harrison, 1987) of the soil organic P pool, whereas more simple organic P forms, such as nucleic acids, sugar phosphates, and phospholipids represent a small proportion (Stewart and Tiessen, 1987). Phytate (inositol hexakisphosphate) is the most abundant inositol phosphate in soil P (Turner et al., 2002). It is the principal storage form of P in seeds of cereals and grains and is introduced to the soil by plant residues and animal manure (Gerke, 2015). The causes for its abundant occurrence are not fully clear. Two main hypotheses have been proposed, viz., the low activity of phytase in soil and the strong binding of phytate to the soil solid phase with its consequent stabilization and accumulation in soil (Gerke, 2015). Use of phytate as a P source depends both on the ability of plants and microbes to produce the enzymes required for hydrolysis and on the bioavailability of phytate. In order to hydrolyze phytate, organisms use various phosphatases, mainly phytase (myo-inositol hexakisphosphate phosphohydrolase) and phosphomonoesterases, e.g., acid phosphatase. The latter category of enzymes hydrolyses lower-order inositol phosphates, but not phytate (Nannipieri et al., 2011).

Arbuscular mycorrhizal fungi (AMF) form symbiotic relationships with the roots of more than 80% of terrestrial plants (Smith and Read, 2008). The main benefit for the plants is enhanced access to and uptake of orthophosphate, possibly in conjunction with an enhanced desorption rate of adsorbed P (Cardoso and Kuyper, 2006). Though it was shown that AMF hyphae display phosphatase activity (Joner and Johansen, 2000), only few studies have addressed the question whether AMF hydrolyze phytate and, as a result, increase plant P uptake. Koide and Kabir (2000) demonstrated that the hyphae of *Glomus intraradices* (now *Rhizophagus intraradices*) utilized phytate and transported the released P to roots. Feng et al. (2003) demonstrated that hyphae acquired P from sodium phytate in the root-free compartment and transferred P to *Trifolium pratense*, but they did not demonstrate hyphal production of phytase.

Some studies assessed acid phosphatase rather than phytase activity (Wang et al., 2013; Zhang et al., 2014). Acid phosphatase hydrolyses only some of the degradation products of phytate, with a lower number of phosphate groups (Kaneko et al., 1990), and only plays an additional role after lower-order inositol phosphates have been formed by phytase (Nannipieri et al., 2011). This hypothesis is supported by the observation that wheat cannot use phytate-P as efficiently as other organic P sources due to lack of phytase, even though it produces acid phosphatase (Richardson et al., 2000).

Within plant species, there is genetic variation in dependency on and responsiveness to AMF (Kaeppler et al., 2000; Gao et al., 2007; Galván et al., 2011; Chu et al., 2013). This variation may be used to breed cultivars that derive more benefit from the mycorrhizal symbiosis. It is not known, however, whether there is also genetic variation among plant cultivars to benefit from mycorrhizal symbioses when the major source of P is phytate.

The aim of our study was to distinguish the roles of AMF-related phytase and acid phosphatase in plant P acquisition from phytate. We applied increasing rates of phytate to a root-free hyphal compartment. To test whether there is an interaction between maize cultivar and AMF species in acquiring P from phytate, we selected an old cultivar and a new hybrid cultivar of maize and two AMF species.

## Materials and Methods

### Soil and Microcosms

A calcareous loamy soil was collected from field plots at the Changping Long-Term Fertilizer Station of China Agricultural University in Beijing, China. The soil contained 17.8 g kg^-1^ organic C, 2.9 mg kg^-1^ Olsen-P, 87.2 mg kg^-1^ N, 155.7 mg kg^-1^ exchangeable K, and had a pH value (in CaCl_2_) of 7.8. The soil was passed through a 2-mm sieve and sterilized by radiation with ^60^Co γ-ray at 10 kGy.

Rhizoboxes (microcosms) were set up as described by Wang et al. (2013). Every box had two compartments (a root compartment and a hyphal compartment), separated by 30 μm nylon mesh through which hyphae but not roots could pass. In the hyphal compartment, a buffer zone of 2 cm width from the mesh window was set up to minimize the influence of roots on the hyphal compartment. Soil was added to the rhizoboxes in the following amounts: 750 g in the root compartment with 20 mg P kg^-1^ [as KH_2_PO_4_; previous research (Wang and Feng, unpublished) had shown extremely poor maize growth without any P fertilizer]; 300 g in the buffer zone without any additional P, and 450 g in the hyphal compartment with 20, 100, and 200 mg P (as calcium phytate; Sigma-Aldrich, St. Louis, MO, USA) kg^-1^ soil. Because of the extremely low available P, we did not include a control without phytate in the hyphal compartment. The other nutrients (kg^-1^ soil) were added uniformly only to the root compartment: 200 mg N (as KNO_3_), 50 mg Mg (as MgSO_4_), 5 mg Zn (as ZnSO_4_), and 2 mg Cu (as CuSO_4_). The nutrients were mixed with the soil before filling of the pots. Three weeks after sowing, another 100 mg N (as KNO_3_) kg^-1^ soil was added to every root compartment.

### Host Plant and Mycorrhizal Fungal Inoculum

Two maize cultivars were used: Huangmaya (HMY), an open-pollinated, early senescing cultivar, which was bred in the 1950s (Chu et al., 2013); and Xianyu335 (XY335), a modern hybrid. The two maize cultivars differ in mycorrhizal responsiveness. HMY is more responsive than XY335 in low-P soil, while the reverse is true when soil Olsen-P is high (X.-X. Wang, unpublished). In each root compartment, two maize seeds (surface-sterilized for 10 min in 10% H_2_O_2_ followed by 3 min in 70% ethanol) were sown; they were thinned to one plant after germination. Soil moisture was kept at 18–20% (w/w, i.e., 70% of water holding capacity) as determined gravimetrically by weighing the pots every 2 days during the experiment and adding tap water when necessary. The glasshouse temperature range was 23–31°C.

The AMF species *Funneliformis mosseae* (formerly *Glomus mosseae*, reference number BGC HEB07B, 1511C0001BGCAM0049) and *Claroideoglomus etunicatum* (formerly *Glomus etunicatum*, BGC HEB07A, 1511C0001BGCAM0048) were used in this experiment. Both were obtained from the Bank of Glomeromycota of China, Institute of Plant Nutrition and Resources, Beijing Academy of Agriculture and Forestry Research. The two AMF species colonized both maize cultivars in previous experiments (unpublished), resulting in a significant growth response. The fungi were propagated in a 5:1 mixture (w/w) of zeolite and river sand with maize for 4 months in a greenhouse. Inoculum consisted of soil containing spores, mycelium, and fine root segments. Forty grams of inoculum was added to each mycorrhizal root compartment, and 40 g of sterilized inoculum (a mixture of both AMF species) was added to non-mycorrhizal root compartments. To minimize differences in microbial communities of mycorrhizal and non-mycorrhizal treatments, 10 mL of AMF-free filtrate from the inoculum was added to each non-mycorrhizal pot, and 10 mL of deionized water was added to each mycorrhizal pot.

### Harvest and Sample Analysis

Plants were harvested 45 days after sowing. At harvest, plant roots were carefully removed from the soil and shaken gently to remove loosely adhering soil. Soils in the root compartment, the buffer compartment and the hyphal compartment were collected separately. The top 2 cm of soil from the hyphal compartment was discarded to eliminate any surface effects. Soil from the buffer section was also discarded. The remainder was mixed in a blender to obtain a uniform matrix for subsequent analyses.

Plants were separated into shoots and roots. The shoots were oven-dried at 105°C for 30 min to cease metabolic activity and then at 72°C for 48 h and finally ground to a fine powder. Roots were washed with deionized water and then preserved at -20°C.

Frozen roots were cut into 1-cm segments and thoroughly mixed. A 0.5-g subsample was cleared with 10% (w/v) KOH at 90°C for 2 h and stained with trypan blue for quantification of mycorrhizal colonization (Trouvelot et al., 1986). Extraradical hyphae were extracted from two 5 g soil sub-samples from the hyphal compartment using the membrane filter technique (Staddon et al., 1999). Hyphal length was assessed using the gridline intercept method at 200× magnification and then converted to hyphal length density [m g^-1^ dry weight (dw) soil]. Shoot P concentration was determined by the standard vanado-molybdate method (Murphy and Riley, 1962), after digestion in a H_2_SO_4_–H_2_O_2_ mixture at 360°C for 2 h.

Soil solution for enzyme assays was obtained by gently shaking about 0.5 g of moist soil with 2 mL deionized water for 1 min. After settling, the suspension was collected for assays and the sediment was dried at 90°C for 24 h to determine dry weight as a reference base.

Phytase activity was assessed according to Richardson et al. (2000): 0.5 mL soil solution was mixed with 2 mL of 30 mM MES [2-(*N*-morpholino) ethanesulfonic acid] buffer (pH 5.5) with 0.5 mL of 2 mM EDTA (ethylenediaminetetraacetic acid) and with 0.5 mL of 20 mM Na-phytate (Sigma, St. Louis, MO, USA). The mixture was incubated for 1 h at 37°C and the reaction terminated by addition of 1 mL 25% trichloroacetic acid (TCA). Solutions were subsequently centrifuged at 12,000 × *g* for 10 min to remove soil particles. To the controls TCA was added prior to incubation. The orthophosphate concentration in the supernatant was determined by measuring absorbance at 882 nm using the molybdenum-blue reaction (Murphy and Riley, 1962).

Acid phosphatase (phosphomonoesterase) activity in hyphal and root compartments was assayed according to Neumann (2006): 0.5 mL soil solution was transferred into 2 mL Eppendorf reaction vials, to which 0.4 mL of 200 mM acetate buffer (pH 5.2) and 0.1 mL of 150 mM substrate [pNPP (*p*-nitrophenylphosphate); Sigma St. Louis, MO, USA] were added. The mixture was incubated for 30 min at 30°C, after which the reaction was terminated by addition of 0.5 mL of 0.5 M NaOH and centrifugation for 10 min at 12,000 × *g* which also removed soil particles. In controls, the substrate was added only after incubation. The concentration of pNPP in the supernatant was measured spectrophotometrically at 405 nm (Joner and Johansen, 2000).

### Experimental Design and Statistical Analysis

Normally, soil total P levels are between 300 and 500 mg kg^-1^ in agricultural soils (Wang et al., 2014). With the fraction of phytate-P in soil being in the range between 30 and 50% (Turner et al., 2002), many agricultural soils would show phytate P-levels between 90 and 250 mg kg^-1^. The experiment was set up in a randomized complete full factorial block design with three factors: (1) phytate—three levels in the hyphal compartment, 20, 100, and 200 mg kg^-1^ P as Ca-phytate (Sigma-Aldrich, St. Louis, MO, USA), which made the amounts we applied similar to those of other experiments; (2) cultivar—two maize cultivars, HMY and XY335; (3) AMF—three treatments, without AMF, and inoculated with *F. mosseae* or *C. etunicatum*. The experiment was carried out with four replicates, giving 72 rhizoboxes in total. The 18 rhizoboxes within a block were arranged randomly in the glasshouse, with the positions re-randomized every week.

Statistical analyses were performed with SPSS software, version 19.0 (SPSS Inc., Chicago, IL, USA). Data met requirements of homogeneity of variance (Levene’s test) except phytase activity and acid phosphatase activity. After log-transformation these data also met the ANOVA assumptions. Three-way analysis of variance was performed to test for significant sources of variation in dependent variables. Where necessary, we report results of ANOVA both including and excluding the non-mycorrhizal treatment. Means were compared with Tukey’s honestly significant differences test at the 5% level of probability. Correlation between variables was tested using Pearson’s correlation coefficient (*P* < 0.05).

## Results

### Enzyme Activities

**FIGURE 1:**
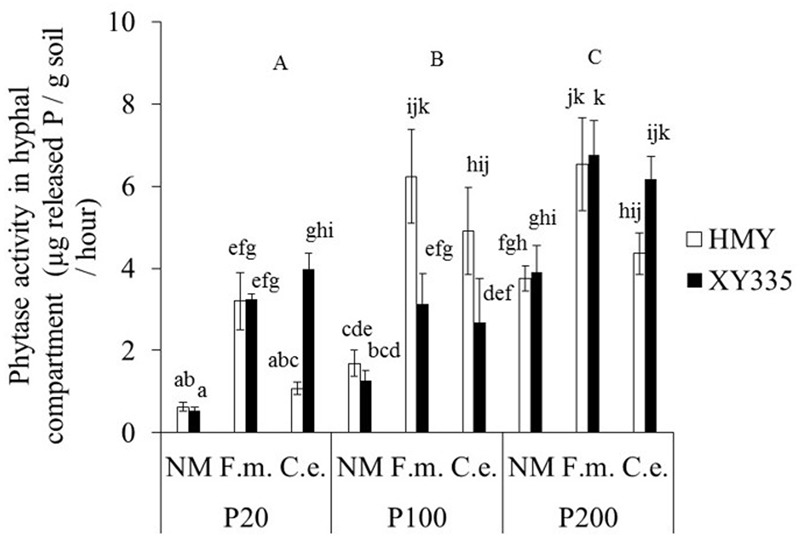
**Phytase activity in the hyphal compartment of two maize cultivars (HMY and XY335) grown with 20, 100, or 200 mg phytate-P kg^-1^ soil in the hyphal compartment.** The plants were non-mycorrhizal (NM) or inoculated with *F. mosseae* (*F.m.*) or *C. etunicatum* (*C.e.*). Bars represent means ± SE (*n* = 4). Treatments with the same lowercase letter are not significantly different (Tukey: *P* < 0.05); uppercase letters refer to main effects of phytate additions.

The effects of phytate addition, cultivar and mycorrhizal treatment on phytase activity in the hyphal compartment were complex: all interactions were significant (**Table 1**). Maize cultivar had no main effect on phytase activity. Phytase activity was significantly higher with 200 mg P kg^-1^ than with 20 and 100 mg P kg^-1^ for XY335, but for HMY phytase activity was significantly higher with 100 and 200 mg P kg^-1^ than with 20 mg P kg^-1^ (**Figure 1**). This result indicated that AMF-mediated changes in phytase activity were also affected by maize cultivar. Phytase activity was around 3% of that of acid phosphatase activity.

**Table 1:** ANOVA results with phytase activity in the hyphal compartment as dependent variable, and phytate addition (P), cultivars (CV), and mycorrhizal treatments (M) as independent variables.

Independent variable	With non-AMF control			Without non-AMF control		
	df	*F*	*P*-value	df	*F*	*P*-value
P	2	98.8	<0.001	2	28.8	<0.001
CV	1	0.004	0.95	1	0.3	0.57
M	2	92.8	<0.001	1	11.5	0.002
P × CV	2	18.0	<0.001	2	21.6	<0.001
P × M	4	9.4	<0.001	2	0.6	0.56
CV × M	2	5.8	0.005	1	9.4	0.004
P × CV × M	4	5.6	0.001	2	4.3	0.02

*The dataset was analyzed twice: once including the non-mycorrhizal control and once excluding this treatment.*

**FIGURE 2:**
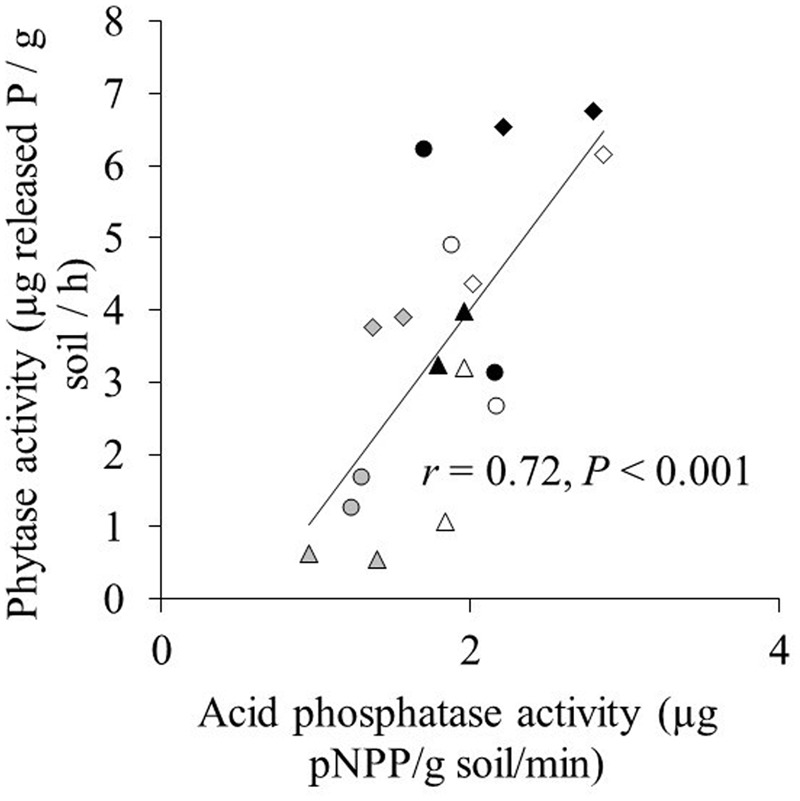
**The relation between acid phosphatase activity and phytase activity in the hyphal compartment.** Triangle, circle, and diamond indicate the 20, 100, and 200 mg phytate-P kg^-1^ soil in hyphal compartment, respectively. Gray, white, and black marks indicate control, *C. etunicatum*, and *F. mosseae* treatment, respectively. Each symbol represents one cultivar and the means of four replicates.

Acid phosphatase activity in both the hyphal and root compartment was significantly affected by phytate addition and cultivar, and their interaction (**Table 2**). Effects of the two AMF species on acid phosphatase activity were similar for both maize cultivars and across the three phytate levels. Acid phosphatase activity in the root compartment and in the hyphal compartment were significantly and positively correlated regardless of whether the non-mycorrhizal treatment was included (*r* = 0.82; *P* < 0.001; **Supplementary Figure S1**) or excluded (*r* = 0.83; *P* < 0.001) in the correlation analysis. Acid phosphatase activity was significantly higher in the root compartment than in the hyphal compartment (**Supplementary Figure S1**). Acid phosphatase was significantly positively correlated with phytase activity (*r* = 0.72; *P* < 0.001; **Figure 2**).

**Table 2:** ANOVA results with acid phosphatase activity in root and hyphal compartments as dependent variable, and phytate addition (P), cultivars (CV), and mycorrhizal treatments (M) as independent variables.

Independent variable	df	Root compartment		Hyphal compartment	
		*F*	*P*-value	*F*	*P*-value
P	2	3.9	0.05	14.1	<0.001
CV	1	11.2	0.001	13.3	0.002
M	1	0.1	0.87	0.04	0.79
P × CV	2	1.7	0.23	4.7	0.04
P × M	2	0.4	0.60	0.2	0.72
CV × M	1	3.4	0.10	0.5	0.47
P × CV × M	2	1.1	0.36	0.6	0.58

*The non-mycorrhizal control was not included in the analysis.*

### Hyphal Length Density and Root Colonization

Hyphal length density was significantly affected by phytate, AMF, and most interactions (**Table 3**). There were no differences between both cultivars. It was about 10% higher at 100 mg kg^-1^ phytate-P (2.7 m g^-1^ soil) than at 20 mg kg^-1^ (2.4 m g^-1^ soil; *P* = 0.014) and at 200 mg kg^-1^ treatments (2.3 m g^-1^ soil; *P* < 0.001; **Supplementary Figure S2**).

**Table 3:** ANOVA results with hyphal length density in the hyphal compartment, and root colonization as dependent variable, and phytate addition (P), cultivars (CV), and mycorrhizal treatments (M) as independent variables, considering the AMF-inoculated treatments only.

	df	Hyphal length density		Root colonization	
		*F*	*P*-value	*F*	*P*-value
P	2	5.3	0.01	0.8	0.47
CV	1	1.5	0.23	10.3	0.003
M	1	34.1	<0.001	17.8	<0.001
P × CV	2	25.4	<0.001	3.6	0.04
P × M	2	10.5	<0.001	0.5	0.63
CV × M	1	0.01	0.98	22.0	<0.001
P × CV × M	2	9.9	<0.001	1.9	0.16

The effect of AMF species on mycorrhizal root colonization was similar for all phytate-P levels, but it was cultivar-dependent (**Table 3**): a larger fraction of the roots of HMY was colonized by *F. mosseae* (56%) compared to *C. etunicatum* (46%), whereas XY335 showed the opposite pattern (**Supplementary Figure S3**).

### Shoot Biomass and Shoot P Content

**FIGURE 3:**
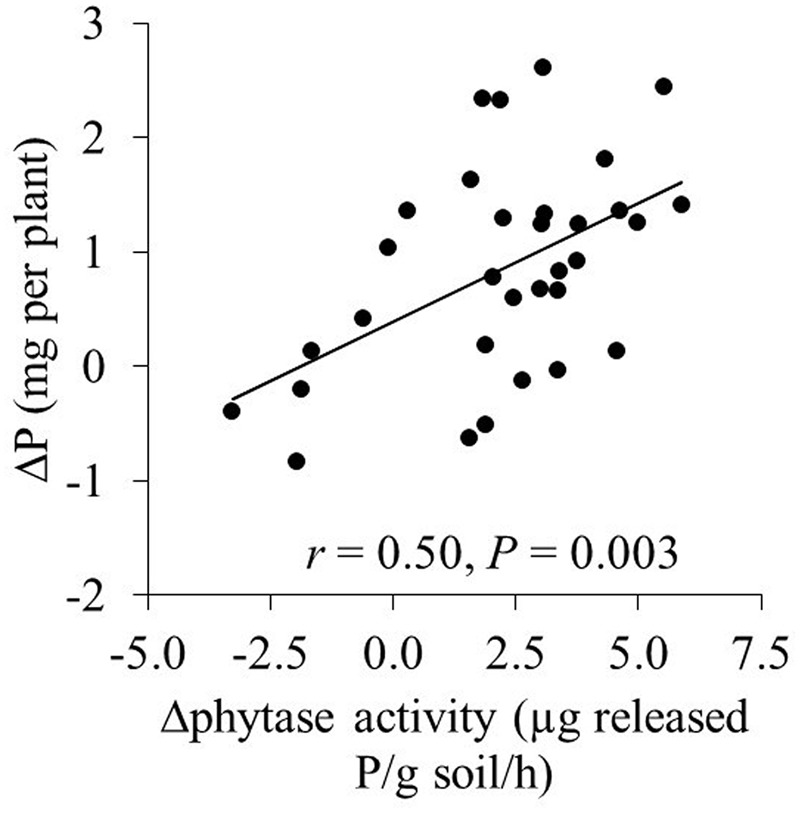
**The relation between the increase (Δphytase) in phytase activity (compared to the addition of 20 mg P kg^-1^) and the increase in P uptake (ΔP) in the mycorrhizal treatments.** Each symbol represents one cultivar and the means of four replicates.

**FIGURE 4:**
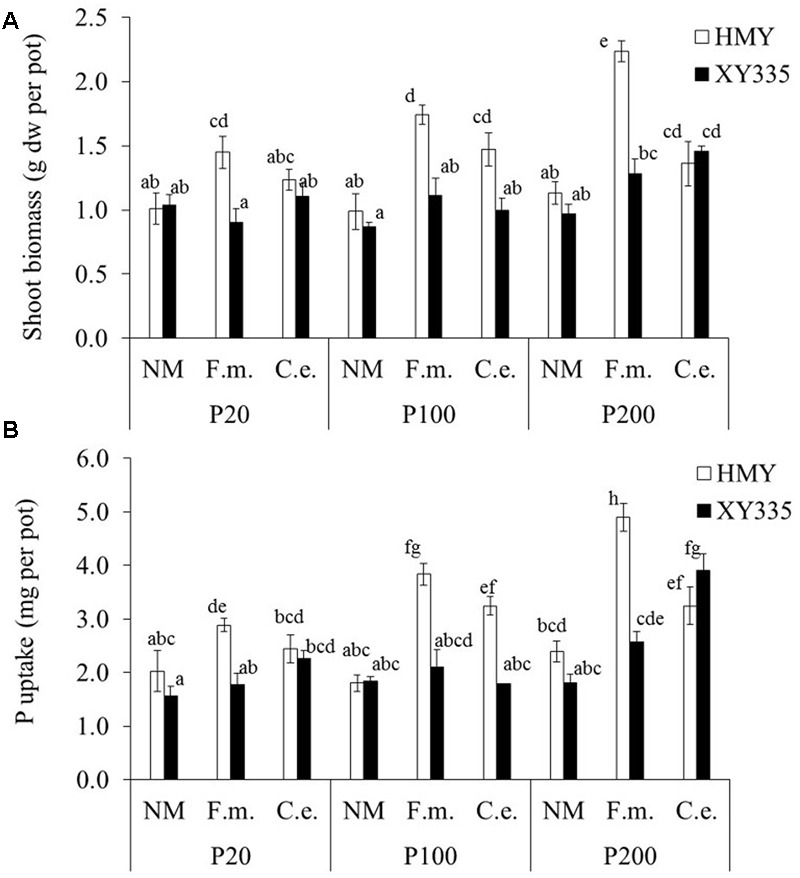
**Shoot biomass (A)** and shoot P content **(B)** of two maize cultivars (HMY and XY335) grown with 20, 100, or 200 mg phytate-P kg^-1^ soil in the hyphal compartment. The plants were non-mycorrhizal (NM) or inoculated with *F. mosseae* (*F.m.*) or *C. etunicatum* (*C.e.*). Bars represent means ± SE (*n* = 4). Bars with the same letter are not significantly different (Tukey: *P* < 0.05).

Shoot biomass and P content of the non-mycorrhizal treatments were not affected by phytate addition (**Figure 4**), confirming that plant roots did not take up any P from phytate added to the hyphal compartment. Shoot biomass and P content of the AMF-inoculated treatments increased with increasing phytate addition in both cultivars (although less strong for XY335; **Figure 4**). The mycorrhizal responsiveness increased with phytate addition for both cultivars. Cultivar HMY responded more strongly to *F. mosseae* than *C. etunicatum*, whereas XY335 tended to show the opposite response (**Table 4** and **Figure 3**).

**Table 4:** ANOVA results with shoot biomass and shoot P content as dependent variables, and phytate addition (P), cultivars (CV), and mycorrhizal treatments (M) as independent variables, with the non-mycorrhizal control included.

Independent variable	df	Biomass		Shoot P content	
		*F*	*P*-value	*F*	*P*-value
P	2	5.8	0.005	27.3	<0.001
CV	1	30.6	<0.001	32.6	<0.001
M	2	20.5	<0.001	21.8	<0.001
P × CV	2	0.2	0.78	4.3	0.02
P × M	4	1.3	0.27	5.5	0.001
CV × M	2	12.5	<0.001	26.3	<0.001
P × CV × M	4	1.5	0.21	5.3	0.001

Phytate addition of 100 and 200 mg P kg^-1^ resulted in average increases of 1 and 2 mg P in shoots, respectively, compared to 20 mg P kg^-1^. In some cases, however, phytate addition did not result in increased P uptake; for example, XY335 did not respond to P addition from 20 to 100 mg kg^-1^. The increase in shoot P content (compared to the treatment with 20 mg phytate-P kg^-1^) correlated significantly and positively with increases in phytase activity in the hyphal compartment (*r* = 0.50; *P* = 0.002; **Figure 2**). Changes in acid phosphatase activity did not correlate with changes in P uptake (*r* = 0.26; *P* = 0.16).

## Discussion

### Enzyme Activities

The lower activity of phytase and acid phosphatase in the hyphal compartment of the non-mycorrhizal control treatment compared to the AMF-inoculated treatments confirms that mycorrhizal fungi or bacteria associated with the mycorrhizal mycelium produce these enzymes (Tarafdar and Claassen, 1988). Our study cannot unequivocally assign enzymatic activity to the fungus, so the contradictory results of transcriptomic studies (that suggest that AMF do not possess the genes for phytase; Tisserant et al., 2012) and earlier experiments (that indicated hydrolysis in an axenic system without contaminating microorganisms; Koide and Kabir, 2000) cannot be resolved. Recent studies have shown that AMF hyphae can promote the growth of phytase-producing bacteria by excreting organic compounds, e.g., carboxylates and sugars, in hyphosphere soil (Zhang et al., 2014, 2016).

Since both phytase and acid phosphatase hydrolyze pNPP (Li et al., 1997), the activity assayed with pNPP as substrate includes the activity of both enzymes (but with phytase making only a minor contribution because of its low activity compared to acid phosphatase). Our observation that phytase activity was only around 3% of acid phosphatase activity is consistent with earlier observations by Hayes et al. (1999), who observed that phytase activity was less than 5% of acid phosphatase activity; and by Barrett-Lennard et al. (1993), who found phytase activity was on average 7% of acid phosphatase activity in excised root segments.

Activities of acid phosphatase and phytase were correlated and were higher after phytate addition to the hyphal compartment (**Figure 2**). A positive correlation between both enzymes was also found for 16 plant species grown at P-deficiency and P-sufficiency (Li et al., 1997). However, whereas increases in phytase activity correlated positively with increased P-uptake by the maize cultivars (**Figure 2**), no significant correlation was found for increases in acid phosphatase and increased P-uptake. This, in combination with the fact that acid phosphatase does not hydrolyze phytate (Nannipieri et al., 2011), suggests that measurement of acid phosphatase provides only limited and indirect information on phytate utilization.

Considering the pH of the soil (7.8), the question may be raised whether it would have been better to assess alkaline phosphatase rather than acid phosphatase activity. In an experiment with the same soil with cotton (Zhang, unpublished data) we assessed both alkaline phosphatase on pH 8.2 and acid phosphatase on pH 5.2. Activities at both pH levels were significantly correlated (*r* = 0.66, *n* = 80; *P* < 0.001) with phosphatase activity at pH 8.2 being around 60% of phosphatase activity at pH 5.2. Moreover, according to Aono et al. (2004) alkaline phosphatase is primarily produced intracellularly and may be important for nutrient exchange at the symbiotic interface rather than for phosphate acquisition from organic sources from the soil.

### Differential Plant Performance

The two maize cultivars tested responded differently to AMF inoculation and phytate supply (**Table 4**). The old cultivar HMY was more responsive to AMF than XY335 (**Figure 3**), which is consistent with a previous study showing higher responsiveness of HMY when grown on a low-P soil (Chu et al., 2013). ANOVA results on phytate, AMF species and cultivar effects on root colonization and hyphal length density (**Table 3**) showed complicated interaction effects, which makes it hard to explain the differential shoot biomass and shoot P content response by the two cultivars mechanistically. This interaction between maize cultivars and AMF species on hyphal P uptake from phytate may imply that breeders need to consider plant variation and fungal functional diversity in breeding efficient maize cultivars using organic P (Rengel and Marschner, 2005; Wolfe et al., 2008).

## Conclusion

Phytate addition in hyphal compartment improved maize growth and P uptake. The increase of plant P uptake was positively correlated with the increase of phytase activity but not with the increase of acid phosphatase activity. Thus, we conclude that phytase is a critical indicator when assessing phytate utilization. Further studies are needed to clarify the mechanisms of the biological turnover of phytate in soils.

## Author Contributions

X-XW designed the study, harvested samples, collected the data, analyzed the data, wrote the first draft, and prepared the manuscript; EH designed the study and prepared the manuscript; GF proposed the research, designed the study, and prepared the manuscript; TK designed the study and prepared the manuscript.

## Conflict of Interest Statement

The authors declare that the research was conducted in the absence of any commercial or financial relationships that could be construed as a potential conflict of interest.

## Supplementary Material

FIGURE S1 **Correlation between acid phosphatase activity in hyphal compartment and root compartment.** Triangle, circle, and square markers indicate the three levels of phytate (20, 100, or 200 mg phytate P kg^-1^), respectively. Gray, white and black refer to the non-mycorrhizal control, *C. etunicatum* and *F. mosseae* inoculated treatments, respectively. Each marker represents one cultivar and the means of four replicates.

FIGURE S2 **Hyphal length density in hyphal compartments of two maize cultivars (HMY and XY335).** P20, P100, and P200 mean 20, 100, and 200 mg phytate-P per kg soil treatment in the hyphal compartment, respectively. The AMF were *F. mosseae* (*F.m.*) and *C. etunicatum* (*C.e*.). Bars represent means ± SE (*n* = 4). Treatments with the same lowercase letter are not significantly different (Tukey: *P* < 0.05); uppercase letters refer to main effects of phytate additions.

FIGURE S3 **Fractional mycorrhizal root colonization for two maize cultivars (HMY and XY335).** P20, P100, and P200 mean 20, 100, and 200 mg phytate-P per kg soil treatment in the hyphal compartment, respectively. The AMF were *F. mosseae* (*F.m*.) and *C. etunicatum* (*C.e.*). Bars represent means ± SE (*n* = 4). Treatments with the same letter are not significantly different (Tukey: *P* < 0.05).
