# Supplementary figures and images for: Phosphate Uptake from Phytate Due to Hyphae-Mediated Phytase Activity by Arbuscular Mycorrhizal Maize
Source: Front Plant Sci. 2017 Apr 28;8:684. doi: 10.3389/fpls.2017.00684 (PMC5408084; doi:10.3389/fpls.2017.00684)

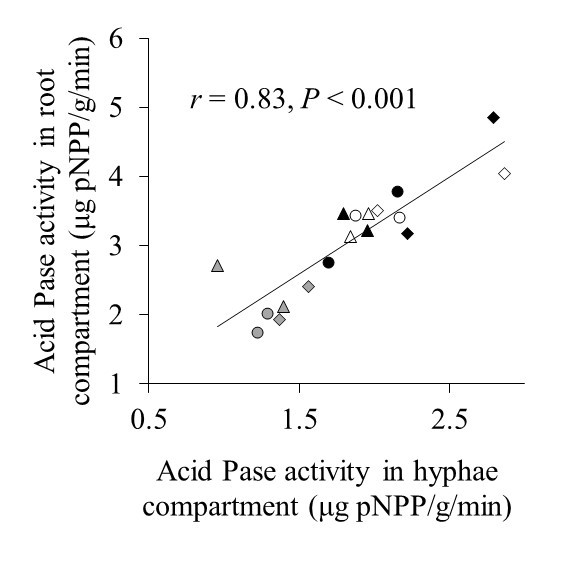

Supplement: FIGURE S1 — Correlation between acid phosphatase activity in hyphal compartment and root compartment. Triangle, circle, and square markers indicate the three levels of phytate (20, 100, or 200 mg phytate P kg-1), respectively. Gray, white and black refer to the non-mycorrhizal control, C. etunicatum and F. mosseae inoculated treatments, respectively. Each marker represents one cultivar and the means of four replicates. [file Image_1.JPEG]

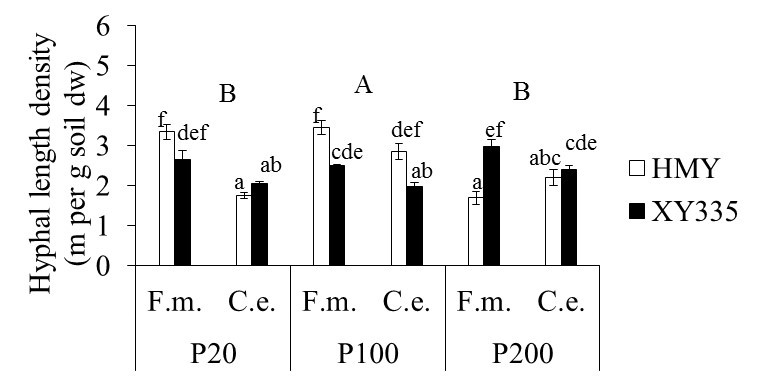

Supplement: FIGURE S2 — Hyphal length density in hyphal compartments of two maize cultivars (HMY and XY335). P20, P100, and P200 mean 20, 100, and 200 mg phytate-P per kg soil treatment in the hyphal compartment, respectively. The AMF were F. mosseae (F.m.) and C. etunicatum (C.e.). Bars represent means ± SE (n = 4). Treatments with the same lowercase letter are not significantly different (Tukey: P < 0.05); uppercase letters refer to main effects of phytate additions. [file Image_2.JPEG]

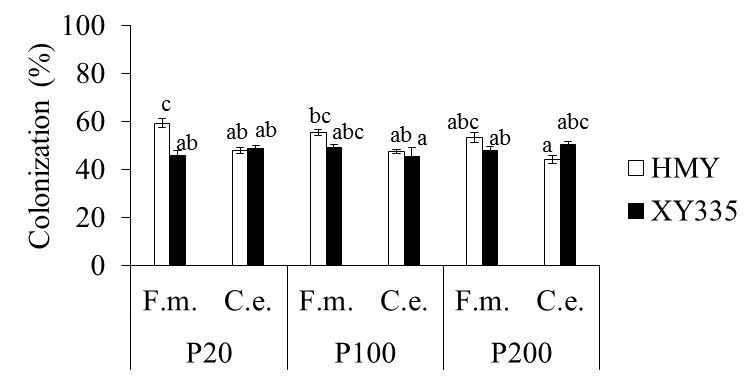

Supplement: FIGURE S3 — Fractional mycorrhizal root colonization for two maize cultivars (HMY and XY335). P20, P100, and P200 mean 20, 100, and 200 mg phytate-P per kg soil treatment in the hyphal compartment, respectively. The AMF were F. mosseae (F.m.) and C. etunicatum (C.e.). Bars represent means ± SE (n = 4). Treatments with the same letter are not significantly different (Tukey: P < 0.05). [file Image_3.JPEG]
